# Evaluation of Euthanasia Methods on Behavioral and Physiological Responses of Newly Hatched Male Layer Chicks

**DOI:** 10.3390/ani11061802

**Published:** 2021-06-17

**Authors:** Xi Wang, Dan Zhao, Allison C. Milby, Gregory S. Archer, E. David Peebles, Shailesh Gurung, Morgan B. Farnell

**Affiliations:** 1 Department of Poultry Science, Texas A&M AgriLife Research, College Station, TX 77843, USA; wangxi@swun.edu.cn (X.W.); dz137@tamu.edu (D.Z.); a.milby@tamu.edu (A.C.M.); garcher@tamu.edu (G.S.A.); sg2353@cornell.edu (S.G.); 2 Department of Poultry Science, Mississippi State University, Starkville, MS 39762, USA; d.peebles@msstate.edu

**Keywords:** carbon dioxide, chicken, euthanasia, low atmospheric pressure, nitrogen, welfare

## Abstract

**Simple Summary:**

Young poultry that are malformed, fail to hatch, or are not economically viable must be humanely killed at the hatchery. Maceration is the predominant method used and is instantaneous and humane. However, it is possible that new methods may be developed which could improve animal welfare and reduce suffering. In this study, we used gases and a vacuum system to kill chicks by reducing available oxygen. We found that using carbon dioxide or the vacuum system resulted in better welfare, as compared to the nitrogen treatment.

**Abstract:**

Newly hatched male layer chicks are considered as “by-products” in the egg industry and must be humanely euthanized at the hatchery. Instantaneous mechanical destruction (maceration) is the predominant euthanasia method applied in poultry hatcheries and is approved by the American Veterinary Medical Association (AVMA). However, maceration is not perceived by the public to be a humane means of euthanasia. The effects of alternative euthanasia methods, including carbon dioxide (CO_2_) or nitrogen (N_2_) inhalation, and a commercial negative pressure stunning system on behavioral and physiological responses of day-of-hatch male layer chicks, were evaluated in a field trial. Chick behaviors, including ataxia, loss of posture, convulsions, cessation of vocalization, and cessation of movement, were monitored. Serum hormones were assessed at the end of each of the alternative euthanasia treatments, including a control group allowed to breathe normal atmospheric air. The N_2_ method induced unconsciousness and death later than the CO_2_ and negative pressure methods, and increased serum corticosterone concentrations of neonatal chicks. Carbon dioxide inhalation increased serotonin concentrations as compared to controls, as well as the N_2_ and the negative pressure methods. The behavioral and physical responses observed in this study suggest that both CO_2_ inhalation and negative pressure stunning can be employed to humanely euthanize neonatal male layer chicks.

## 1. Introduction

Male layers, pipped embryos, and hatched chicks with lethal deformities must be humanely euthanized on day-of-hatch. In 2018, the U. S. egg industry owned over 391.3 million laying hens [1], which means a similar number of male layer chicks were hatched as a by-product. *In ovo* sex determination and embryo euthanasia have been proposed to avoid neonatal culling. However, these methods will require high accuracy and no negative impacts on hatchability and performance, which limits the application of embryo euthanasia [2,3,4]. Fluorescence spectroscopy technology has enabled sex determination at 3.5 days of incubation, but only has a 93% accuracy [5]. Male chicks can be distinguished via feather, vent, and color sexing techniques on day-of-hatch before they are humanely euthanized. A hyperspectral imaging system has the capability of differentiating the gender of layer line birds having gender specific feather coloring [6]. Although this method has a 97% accuracy, it can only be applied to 11-to-14-day-old embryos [6]. However, an embryo’s ability to experience pain develops stepwise beginning of day seven of embryogenesis [7], with full brain maturity being achieved by day 13 [8]. Stress and welfare issues cannot be completely avoided through embryo euthanasia with currently available technology.

The American Veterinary Medical Association (AVMA) recommends maceration for euthanasia of neonates and embryos that have completed 80% of embryogenesis [9]. Maceration, with rotating blades or projections, is predominantly applied at hatcheries as a humane killing method of chicks within 72 h post hatch, which causes minimal pain and distress because of its rapid physical disruption of the brain [9]. Although maceration is believed to be comparable to cervical dislocation, it can be negatively perceived by some consumers. Therefore, primary breeders and hatcheries are exploring alternative euthanasia methods.

Carbon dioxide (CO_2_), nitrogen (N_2_), argon (Ar), and their combinations are approved by the AVMA for euthanizing poultry [9]. Carbon dioxide, at a concentration ranging from 40 to 45%, is capable of humanely killing broilers at 4 to 6 weeks of age [10,11,12]. Compared to mature birds, newly hatched chicks have a higher tolerance to hypoxia, which lasts up to eight days post hatch [13]. Before homoeothermic metabolism develops at eight days of age, chicks are poikilothermic, which reduces their oxidative metabolic demands when they become hypoxic [13]. Gurung, et al. [14] observed in a previous study that male hatchlings subjected to 25 and 50% CO_2_ recovered after they were removed from the experimental chamber in which they were held [14]. A higher concentration of CO_2_ at 75% was needed for successful chick euthanasia [14]. Baker, et al. [15,16] reported that immersion into a 90 to 100% CO_2_ environment resulted in the shortest time to insensibility and death and decreased the duration and frequency of distress behaviors compared to gradual displacement rates. Nitrogen and argon, which are inert and non-toxic gases, can displace oxygen (O_2_) in the atmosphere, causing birds to experience a loss of consciousness and hypoxia [14,17]. However, O_2_ concentrations should be less than 2% when using an inert gas to cause hypoxia [9]. Limited research has been conducted to evaluate the efficacy of N_2_ euthanasia on day-old chick euthanasia [14].

Gradual decompression of the atmosphere in a chamber (low atmospheric pressure stunning; LAPS™) can reduce O_2_ tension to achieve a progressive anoxia for poultry [18,19]. A LAPS™ system has been applied in broiler production as an alternative to electrical stunning at slaughter [20,21]. Behavioral response and brain activity observations conducted on broilers suggest that the LAPS™ method does not induce any escape behaviors and has the potential to improve the welfare of poultry at slaughter [13,22]. The current report is the first to evaluate the effects of this commercial stunning system when employed for the euthanasia of newly hatched chicks.

Our laboratory has evaluated alternative euthanasia methods for adult layer hens [23,24] and neonatal chicks [14] using experimental chambers. In a previous study in our laboratory, the effects of CO_2_, N_2_, and a negative pressure treatment were investigated. Carbon dioxide inhalation was found to be statistically faster in inducing the cessation of movement of neonatal chicks than the N_2_ and negative pressure methods. However, the CO_2_, N_2_, and negative pressure methods had the potential to improve the welfare of chicks because the serotonin levels of the chicks in those treatments were higher than those in a control treatment that could breathe normal atmospheric air [14]. In the current study, the effects of these euthanasia methods on the behavioral and physiological responses of newly hatched male layers were compared in a commercial LAPS™ system.

## 2. Materials and Methods

### 2.1. Experimental Design

The experimental protocol was approved by the Texas A&M University Institutional Animal Care and Use Committee protocol (IACUC-2016-0200). A completely randomized experimental design was applied to investigate the potential effects of various euthanasia methods on chick behavioral and physiological responses. The experiment tested normal atmospheric air (control), CO_2_, and N_2_ inhalation treatments, as well as a commercial negative pressure method treatment (LAPS™, Technocatch LLC., Kosciusko, MS, USA). A total of 480 male layer hatchlings were randomly divided into 16 replicate baskets (30 chicks/basket) on a two-day period. Each treatment was replicated four times on two different days (two replicates/day).

### 2.2. Euthanasia Procedure

The field trial was conducted in Kosciusko, MS using a 30′ gooseneck trailer equipped with a customized gas vaporizer and air mixing system. This equipment provided approximately 1019 standard L/min of breathing quality air, N_2_ or CO_2_. For each gas treatment, a 300-gallon (1136 L) propane tank was pre-charged to 120 PSI, providing 2749 gallons (10,406 L) of gas at atmospheric pressure at the beginning of each treatment. The system continued to supply gas after the initial discharge of the storage tank. For each replicate of the euthanasia study, 30 day-of-hatch layer chicks received one of the respective treatments in a commercial vacuum chamber (volume of 3880 L; Technocatch LLC). Atmospheric air, CO_2_ or N_2_ was added to the chamber according to the treatment setting. A vent plug (2.54 cm diameter) was removed to allow these gases to escape and to prevent pressurization of the vessel. Chick behaviors were monitored by an infrared camera system (Sony, New York City, NY, USA). When chicks stopped movement for one minute, they were immediately removed for blood sampling. Since chicks in the control group were alive during the treatment, they were humanely euthanized by decapitation. Chick death was confirmed by absence of nictitating membrane and bipedal reflexes. Blood samples were collected via post-mortem cardiac puncture and stored on ice. After 24 h of clotting time on wet ice, samples were centrifuged at 4819 to 6238 g for 10 min, using a microcentrifuge (10,000 RPM, RevSpin, Revolutionary Science, Shafer, MN, USA) for serum separation. Serum samples were stored at −80 °C prior to ELISA analysis.

### 2.3. Behavior Determination

A video track of the behaviors of chicks in each treatment replicate group was recorded. Video recording began before treatment application and ended one minute after cessation of movement of all chicks. During euthanasia, all chicks demonstrated behaviors including ataxia, loss of posture, and convulsions, except for those belonging to the control group. According to the behavioral descriptions provided by Mackie and McKeegan [22], ataxia was defined as “apparent dizziness, staggering, and swaying of the body”. Loss of posture was recorded when chicks were unable to maintain or regain a controlled posture [25]. Convulsions in poultry have been defined as severe wing flapping with neck or leg tensing, and uncontrolled muscle movements [10]. Since chicks in the same treatment group exhibited ataxia, loss of posture, and convulsions at different points in time, latencies between the first chick and the last chick demonstrating the defined behavior were recorded at the start and the end of each behavior, respectively. Behavioral duration (seconds) in that group of birds was calculated by deducting the start of the behavior from the end of that behavior. Cessations of vocalization and movement were also evaluated.

### 2.4. Stress Physiology

To evaluate the stress status of chicks, serum corticosterone and serotonin concentrations were examined by ELISA. Serum samples from five randomly selected chicks out of the 30 in each treatment replication (5 chicks × 4 replications = 20 chicks/treatment) were used for serum corticosterone concentration analysis using a commercial kit (ADI-901-097, Enzo Life Sciences, Farmingdale, NY, USA). Serum samples were diluted two-fold to assure that corticosterone concentrations fell in the middle range of the standard curve (ADI-901-097, Enzo Life Sciences). Another five serum samples were randomly selected (5 chicks × 4 replications = 20 chicks/treatment) for the analysis of serotonin concentrations, also using a commercial kit (ADI-900-175, Enzo Life Sciences). According to the manufacturer’s instructions, final concentrations were determined utilizing a four-parameter logistic curve program (Elisakit, Pty Ltd., Melbourne, Australia). All serum samples were analyzed in duplicate.

### 2.5. Statistical Analysis

Statistical analysis was performed using the GLM procedure of SAS 9.4; (Inst. Inc, Cary, NC, USA) [26]. The effects of euthanasia treatment on stress hormone concentrations and chick behaviors were analyzed by one-way ANOVA. If significant global treatment effects were observed, comparisons among treatment means were determined using Fisher’s LSD. The significance level was set as 0.05. Since birds in the control treatment (atmospheric air) were alive throughout the experiment, only behavioral data from the CO_2_, N_2_, and negative pressure treatments were analyzed and compared.

## 3. Results and Discussion

### 3.1. Behavioral Observations

The following consecutive behaviors were observed as birds were euthanized: ataxia, loss of posture, convulsions, and cessations of vocalization and movement. Behavioral latencies and the durations of N_2_, CO_2_, and negative pressure treatment needed to achieve chick euthanasia are presented in Table 1.

The N_2_ treatment resulted in a delay in unconsciousness and death, when compared to the CO_2_ and negative pressure methods. The N_2_ treated chicks took longer to exhibit ataxia, loss of posture, and convulsions, and to cease vocalization and movement, in comparison to chicks treated with CO_2_ or the negative pressure treatment (*p* < 0.001 for all behaviors). When considered as a whole group, the length of time between the first bird and the last bird to exhibit ataxia, loss of posture, and convulsions in the N_2_ treatment were longer than those belonging to the CO_2_ and negative pressure groups (*p* ≤ 0.001 for all behaviors). Similar findings were reported in a previous laboratory study by Gurung, et al. [14], in which chicks treated with N_2_ were the last ones to lose posture and to cease movement. Nitrogen is an odorless, colorless, and tasteless inert gas which comprises 78% of atmospheric air. By displacing O_2_ in the air, N_2_ can cause hypoxia and death. The AVMA [9] recommends less than a 2–3% O_2_ concentration to euthanize animals via inert gas inhalation. Nitrogen has a lower density as compared to air and CO_2_ (1.17, 1.20, and 1.84 kg/m^3^ at 20 °C and 101.3 kPa, respectively). Because of this property, N_2_ takes longer to reach a lethal concentration, as compared to CO_2_, and results in an extended time until death.

Both CO_2_ inhalation and negative pressure treatments induced the death of chicks in a range of 341.0 to 356.5 s, which were significantly faster than the N_2_ treatment (631 s). Furthermore, chicks treated with CO_2_ started convulsing (84.8 s) and stopped vocalizing (94.3 s) sooner than those in the other two euthanasia treatments (*p* < 0.05 for both behaviors). Convulsions associated with severe wing flapping vigorous movements were observed in birds subjected to the gas mixtures [27]. Electroencephalogram (EEG) analysis has indicated that a form of consciousness cannot be excluded during a period of anoxic wing flapping [28], which will cause a significant welfare issue. Vocalization is another important welfare assessment, which reveals a distinctive state of an animal that may occur spontaneously or in response to an external event [29,30]. Acoustic features of layer vocalizations can be used to distinguish physical stress caused by temperature and by mental stress in association with fear [31]. In the present study, the end of vocalizations in layer chicks might be associated with the end of mental stress (fear). Carbon dioxide is denser than air and N_2_, which allows it to sink to the bottom of the chamber, whereas N_2_ mixes with air. Carbon dioxide inhalation can cause anesthesia by lowering the pH of cerebrospinal fluid before animal euthanasia [32,33]. Upon gradual accumulation at the bottom of the chamber, CO_2_ can anesthetize young chicks and terminate their convulsions and vocalizations sooner.

The LAPS™ system (negative pressure method) has been extensively documented to benefit the well-being of pre-slaughter broilers. The LAPS™ system has been reported to induce early high amplitude slow waves of EGG signaling, indicating a loss of consciousness [12], reduce aversive behavioral responses, decrease acute heart elevation during the conscious period [19], and cause limited pathological changes by the decompression and the recompression process [34]. These current data suggest that the commercial negative pressure method is a humane method for culling male layer chicks. Neonates treated with negative pressure experienced ataxia, loss of posture, and cessation of movement over a period of time similar to that of chicks euthanized by CO_2_ inhalation (Table 1). In coincidence with a previous broiler study [19], layer chicks did not exhibit any escape behaviors during the negative pressure treatment. Loss of posture occurs simultaneously after the suppression of electrical activity of the brain, and it can be used as an indication of unconsciousness in birds [10]. Negative pressure-treated chicks lost posture within an average of 58.8 s, which was consistent with that of fast-growing broilers reported in a study by Martin, et al. [12]. Additionally, EEG and cardiac measures support the contention that the LAPS™ system can cause 35-day-old broilers to lose consciousness within 60 s [12]. However, the EEG and cardiac variables of neonatal chicks should be monitored in future trials.

### 3.2. Serum Corticosterone

**Figure 1:**
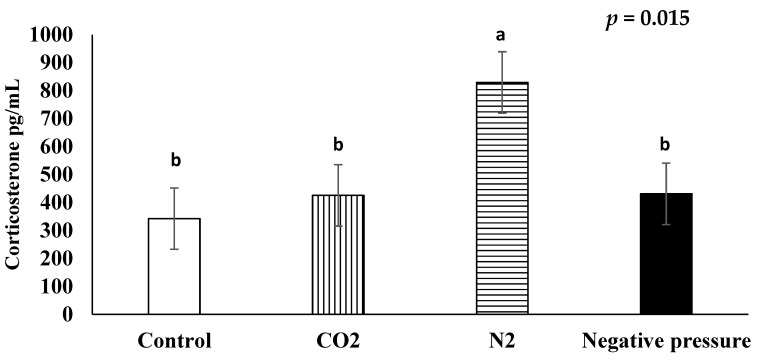
Effects of euthanasia methods on serum corticosterone concentration of newly hatched male layers. ^a,b^ means not sharing the same letter are considered different when the *p*-value is ≤ 0.05. Each treatment was replicated four times and five serum samples per replication were randomly selected (*n* = 20 chicks/treatment). Serum samples from untreated control chicks were taken after decapitation.

The average serum corticosterone concentrations in chicks subjected to atmospheric air, CO_2_, N_2_, and negative pressure treatments were 342.5 pg/mL, 425.7 pg/mL, 829.2 pg/mL, and 430.9 pg/mL, respectively (Figure 1). The serum corticosterone concentrations of chicks in the N_2_ treatment were higher than those in all the other treatments (*p* < 0.05); whereas the serum corticosterone concentrations of the birds in the CO_2_ and negative pressure treatments were not higher than those in the atmospheric air control group.

Serum corticosterone concentration is considered as a primary stress indicator in poultry [35]. When birds perceive environmental stimuli as threatening, the stress response activates the hypothalamic–pituitary–adrenal axis, which results inthe release of corticosterone [36]. In the current field study, the atmospheric air-treated control birds exhibited an average serum corticosterone concentration of 342.5 pg/mL. This level of corticosterone was numerically lower than that in broilers raised in a commercial house equipped with black curtains and whose circulating corticosterone concentrations ranged from 647 to 762 pg/mL [37]. Laying hens raised in commercial cages (2 birds/1.12 m^2^) have exhibited a range of 610 to 1410 pg/mL of plasma corticosterone [38]. The commercial chamber in this field study provided a large dark space (3.89 m^3^), which may have mitigated chick stress and anxiety. Martin, et al. [39] conducted a trial to reveal the effects of light on chicken responses to LAPS™ and reported the darkness reduced the time to unconsciousness as compared to the illumination during LAPS™ application [39]. Furthermore, a mice study indicated a darkened chamber reduced anxiety behaviors [40].

Hypoxia in poultry resulting from their exposure to N_2_ gas has been approved by the AVMA [41]. However, in this study N_2_ inhalation increased the serum corticosterone concentrations of neonatal chicks, as it took longer to fill the chamber with N_2_ to achieve a lethal dosage. The chicks could experience anxiety and discomfort during this long process, which resulted in increased stress hormone levels. Likewise, the AVMA has shown a concern that a hypoxic condition prior to unconsciousness would compromise the welfare of animals. The effects of N_2_ on the birds may be delayed because they did not start exhibiting ataxia and other adverse behaviors until 104.3 s after N_2_ exposure. This contrasts with birds in the CO_2_ group (22.8 s) and in those belonging to the negative pressure group (29.3 s). The stress response in poultry to N_2_ hypoxia is dependent on the application method. When neonatal chicks have been subjected to N_2_ in a smaller chamber [14], or when spent hens were subjected to N_2_ inhalation [23], corticosterone concentrations were shown not to increase. A fast fill rate or prefilling N_2_ in an enclosed container might be the key to an effective and humane euthanasia method for neonates. Broilers that freely entered a chamber containing less than 2% O_2_ and greater than 90% argon exhibited fewer headshakes and gasping than that of the prefilled chamber with 30% CO_2_ [10]. Nitrogen immersion in a 100% pre-charged chamber may mitigate the stress of hypoxia without causing an increase in circulating corticosterone concentrations.

Neither CO_2_ inhalation nor the application of negative pressure statistically increased the serum corticosterone concentrations of chicks in comparison to those belonging to the atmospheric air control group. This would suggest that both could be considered as humane methods to euthanize day-old chicks. In this field study, CO_2_ filled the commercial chamber gradually, as CO_2_ exposure at a high concentration is known to cause pain due to the formation of carbonic acid in the respiratory and ocular membranes [9]. In a human study, 36 out of 40 participants reported sensations of breathlessness when subjected to 50% levels of CO_2_ [42]. This adverse sensation is due to rapid respiratory motor activity caused by the activation of vascular chemoreceptors in response to hypercapnia caused by CO_2_ [43]. Inducing unconsciousness by a low concentration of CO_2_ is important to eliminate the potential discomfort of rapid breathing and breathlessness. Gradually increasing CO_2_ concentration (20% of chamber volume per minute) is required by the European Union [44] to ensure welfare and to limit pain in animals. However, there is currently no flow rate requirement for the use of CO_2_ in poultry according to 2020 AVMA euthanasia guidelines [9]. There is concern that the use of gradual fill methods may increase the distress of animals before CO_2_ reaches a level to cause unconsciousness. A study in which mice were used, demonstrated that a flow rate between 10 and 30% of chamber volume/minute caused dyspnea, in which deep and quick breathing, and a loss of the bipedal withdrawal reflex resulted [45]. A very low flow displacement rate (10% volume/minute) prolonged the duration of stressful behaviors in rats [46]. Similarly, the use of a higher flow displacement rate (100% volume/minute) increased agitation in the rats [46]. It is suggested that an optimal CO_2_ flow rate should be determined to mitigate the potential distress of chicks prior to unconsciousness.

The present study generated the first report concerning the utilization of LAPS™ to euthanize newly hatched chicks. Serum corticosterone concentrations of the birds were not elevated by the negative pressure treatment when compared to those in the atmospheric air control group, which suggests that LAPS™ could be considered as a humane euthanasia method for neonatal chicks. Rather than electrical stunning methods, the LAPS™ system has been applied in broiler slaughter plants as a humane stunning system, and as a means by which to result in a decrease of the serum corticosterone concentrations in broilers [18]. Immature animals are considered to be more tolerant to hypoxia, which requires a long period of decompression for respiration cessation [41]. However, LAPS™-stunned broilers lost posture by 64.9 ± 6.09 s after its application [18]. This is similar to the length of time (58.8 ± 7.7 s) that the first chick lost posture in the present study. The LAPS™ system may have caused hypoxia within a short period of time and enabled chicks to lose consciousness without affecting their circulating corticosterone concentrations. Additionally, the LAPS™ system may provide a stable and low atmospheric pressure, regardless of the number of birds that are subjected to treatment [21].

### 3.3. Serum Serotonin

**Figure 2:**
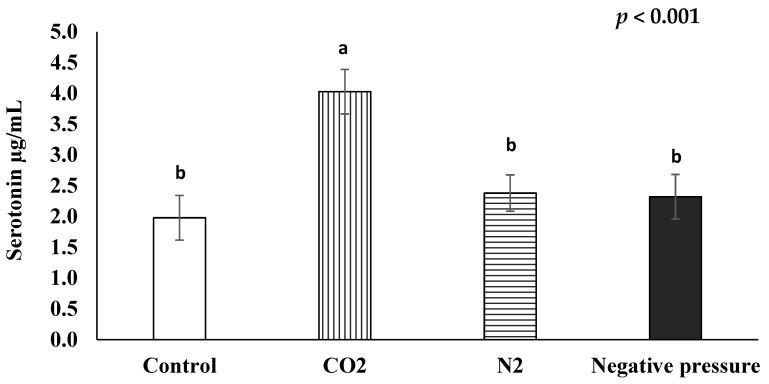
Effects of euthanasia methods on serum serotonin concentration of newly hatched male layers ^a,b^ means not sharing the same letter are considered different when the *p*-value is ≤ 0.05. Each treatment was replicated four times and five serum samples per replication were randomly selected (*n* = 20 chicks/treatment). Serum samples from untreated control chicks were taken after decapitation.

The average serotonin concentrations of chicks subjected to the control, CO_2_, N_2_, and negative pressure treatments were 1.98 µg/mL, 4.03 µg/mL, 2.38 µg/mL, and 2.32 µg/mL, respectively (Figure 2). The CO_2_ treatment significantly (*p* < 0.05) increased the serum serotonin levels of day-old chicks, as compared to those in the other treatment groups.

Serotonin (5-hydroxytryptamine) is mainly produced by the enteric nervous system in the gastrointestinal tract [47] and the central nervous system in the brainstem [48]. It can act as a hormone, a neurotransmitter, and as a mitogen for animals [49]. In poultry, increased serotonin expression is linked to decreased fear-related behaviors [50]. Neonatal chicks with a genotype that leads to a high expression of the serotonin transporter are more resilient in fear tests [51]. Serum serotonin concentrations have been reported to be inversely correlated to corticosterone concentrations in spent hens [23] and in rats [52]. In the present study, chicks treated with CO_2_ exhibited higher serum serotonin concentrations than the birds in the other treatment groups, including the control group. This suggests that they may have experienced less fear than those in the other treatment groups. The increase in serum serotonin concentrations may be also related to the response of the central nervous system to high levels of CO_2_. When animals are exposed to a high level of CO_2_, their brain will release serotonin for regulation of the arousal response and a subsequent increase in autonomic breathing rate [53]. Neurons that synthesize serotonin are sensitive to CO_2_ in situ and a subset of serotonin neurons can be excited by arterial hypercapnia [54]. Moreover, in a rat model, it has been demonstrated that this ventilator response of acute intermittent hypercapnic-hypoxia can be dampened by the blocking of serotonin receptors [55]. However, more research is needed to better understand the role of changes in avian serum serotonin concentrations during euthanasia.

It should be noted that only 30 chicks were placed per replicate of each treatment in the present study. The commercial LAPS™ chamber would allow many more birds to be euthanized than we used in our trials, but we tried to limit the number of animals used and thereby reduce the potential for suffering. The vacuum system can provide a stable low atmospheric pressure, regardless of bird number [21]. However, when considering the volume of birds, the lethal dose of CO_2_ or N_2_ may be achieved sooner if more birds are placed, which will shorten the time to unconsciousness and death. The bird number and container size should be further considered when evaluating gas treatments as euthanasia methods.

One major inadequacy of this project was the inability to compare these euthanasia methods to maceration. Unfortunately, the equipment is not portable and the method itself prevents collecting intact tissues and videos to make a proper assessment. It should be noted that while maceration may not be perceived as aesthetically pleasing, the method is instantaneous, humane and an efficient euthanasia method for embryos and neonatal chicks.

## 4. Conclusions

Overall, these data suggest that CO_2_ inhalation and the negative pressure method can humanely euthanize neonatal chicks, as indicated by the behavioral and hormonal observations. Applying N_2_ in a commercial chamber increased serum corticosterone concentration and prolonged the time to death of layer chicks. A faster fill rate of N_2_ or a prefilled chamber may reduce the time necessary to achieve chick euthanasia and to mitigate the distress of hypoxia.

## Figures and Tables

**Table 1:** Effects of euthanasia methods on behaviors of newly hatched male layers.

	Ataxia			Loss of Posture			Convulsions			Cessation	
Euthanasia Method	Start	End	Duration	Start	End	Duration	Start	End	Duration	Vocalization	Movement
CO_2_	22.8 ^b^	74.8 ^b^	53.5 ^b^	50.8 ^b^	82.0 ^b^	31.3 ^b^	84.8 ^c^	284.3 ^b^	199.5 ^b^	94.3 ^c^	356.5 ^b^
N_2_	104.3 ^a^	270.8 ^a^	166.5 ^a^	147.3 ^a^	252.0 ^a^	104.8 ^a^	306.8 ^a^	620.0 ^a^	313.25 ^a^	576.8 ^a^	631.3 ^a^
Negative pressure	29.3 ^b^	75.0 ^b^	45.8 ^b^	58.8 ^b^	101.3 ^b^	42.5 ^b^	156.5 ^b^	315.3 ^b^	158.8 ^b^	288.8 ^b^	341.0 ^b^
SEM	10.24	13.38	13.26	7.7	14.45	9.78	15.03	19.79	14.83	15.18	29.43
*p*-value	<0.001	<0.001	<0.001	<0.001	<0.001	0.001	<0.001	<0.001	<0.001	<0.001	<0.001

^a–c^ means not sharing the same superscript are considered different when the *p*-value is ≤ 0.05. SEM: standard error of mean. Behavioral responses were monitored as a group of 30 treated chicks. Latencies (seconds) of the first chick and the last chick demonstrating the defined behavior were recorded at the start and the end of that behavior, respectively. Duration (seconds) of the behavior was calculated by deducting the start from the end of that behavior. Each treatment was replicated four times (*n* = 4 replications/treatment).

## Data Availability

Data sharing is not applicable to this article.
